# Age differences in susceptibility to stroke-related health misinformation on social media: the role of prior experience and corrective feedback

**DOI:** 10.3389/fpubh.2026.1850524

**Published:** 2026-06-18

**Authors:** Hongkai Li, Xingyun Liu, Hanshu Zhang

**Affiliations:** 1 Department of Neurosurgery, Wuhan Fourth Hospital, Wuhan, China; 2 School of psychology, Central China Normal University, Wuhan, Hubei, China

**Keywords:** aging decision making, misinformation, misinformation judgment, prevalence effect, social media

## Abstract

**Background and objective:**

The rapid dissemination of complex health-related misinformation poses a critical threat to public health management and healthy lifestyles. To address this infodemic, our current research applied Signal Detection Theory to examine the joint impacts of age, prior stroke-related experience, and information prevalence on individuals' susceptibility to stroke-related misinformation on social media.

**Methods and results:**

In Experiment 1, participants from two diverse age groups evaluated stroke-related statements sourced from social media. Results indicated that no main age effect on the judgment criterion, nor did information prevalence significantly alter these criteria. Notably, a critical interaction emerged: younger adults with prior stroke-related experience (e.g., through family members) exhibited heightened misinformation susceptibility. They tended to adopt more liberal criteria, making them paradoxically more likely to accept misinformation as factual. Experiment 2 showed that providing immediate corrective feedback was associated with more conservative judgment criterion, particularly among those with prior stroke-related experience. Experiment 3 provided preliminary evidence that repeated exposure to corrective feedback was associated improved task-specific discriminability at a 1-week follow-up.

**Conclusion:**

Our research suggests that while information prevalence does not inherently dictate individuals' judgment, personal experience can unexpectedly increase vulnerability to online health misinformation. Overall, these findings suggest that corrective feedback may help calibrate judgment criterion in online health-information evaluation, although its causal and long-term effects require further testing with longer follow-up intervals.

## 1 Introduction

The rapid and unfettered dissemination of content on digital social media platforms has long fueled a widespread “infodemic” (1), fundamentally challenging public health management. Among the various types of unverified content circulating online, health-related misinformation, defined as claims about health that are false or unsupported by insufficient scientific evidence, is arguably the most impactful, as it directly targets broad public concerns (2). Misleading claims regarding critical topics such as vaccines, COVID-19, HPV (human papillomavirus) and the Zika virus can easily serve as a misleading source of health information for many (3, 4). Recently, this pre-existing public health crisis has been further exacerbated by the rapid advancement of generative AI (GenAI) technologies. The growing sophistication of GenAI in producing highly persuasive, human-like text has made it increasingly difficult for individuals to distinguish between authentic medical facts and fabricated misinformation, thereby amplifying the threat to digital health literacy (5, 6).

Despite efforts to combat health-related misinformation on social media, such as careful dissemination of medical research, targeted expert fact-checking, or corrective responses (7), the overwhelming prevalence of online misinformation continues to pose a significant challenge. In this way, navigating this complex and distorted digital environment constantly demands evaluating the authenticity of unverified claims. Previous cognitive research indicates that the prevalence of a target in the environment can implicitly shape human judgment (8). Specifically, studies in perceptual decision making have shown that when targets are rare, individuals tend to shift their decision thresholds and adopt more conservative criterion [i.e., Low Prevalence Effect, LPE; (9)]. While this phenomenon has been fully developed in basic visual search tasks involving abstract stimuli (10, 11) and real-world objects (12) in various search environments (13, 14), little is known about how such imbalances in information prevalence affect the processing of complex textual health information. Understanding this is critical for public health: when algorithms amplify health-related misinformation on social media, authentic medical facts are often reduced to low-prevalence targets, potentially compromising decision makers' evaluation standards and increasing their misinformation susceptibility (15). Therefore, the current study aimed to examine how variations in information prevalence impact individuals' judgment criterion during a text-based misinformation fact-checking task.

To investigate these influential factors in a high-stakes public health context, our current study focuses on the evaluation of stroke-related misinformation. In China, stroke is the primary cause of disability-adjusted life years, imposing a significant strain on healthcare systems (16, 17). Despite the critical significance of stroke awareness in preventive measures and acute interventions, there was a deficiency in understanding risk factors and warning symptoms among the public (18). Given that prompt medical response is crucial in acute stroke management to reduce cerebral damage and improve recovery, stroke-related misinformation on social media may severely delay emergency assistance (19). Identifying the populations that are particularly vulnerable to such misinformation is therefore a public health priority.

Traditionally, adults entering age ranges with increased stroke-vulnerable relevance may face distinct challenges when evaluating online health information (20–22). In addition to a lack of digital literacy and health literacy (23), this vulnerability is often attributed to age-related declines in cognitive abilities (24, 25), selection of decision strategies (26), brain activation (27, 28), and sensitivity to the weighting of gains and losses (29, 30). Specifically, when it comes to the judgment criterion, Devine et al. (31) found that while cautious responding by older adults may be disadvantageous in certain cognitive domains, it could serve as a protection against biased evaluation. However, susceptibility to health misinformation is not solely dictated by age. Intrinsic motivations and personal relevance, such as having a personal or family history of a specific disease, can also alter how individuals process health claims (32). Therefore, our current research also examined whether such experience protects individuals or paradoxically increases their misinformation susceptibility across different age groups.

To mitigate the misinformation susceptibility, public health interventions often rely on corrective responses or expert fact-checking on social media platforms (33, 34). While previous research on prevalence effect suggested that the presence or absence of performance feedback can fundamentally shift individual's decision criterion (35, 36), its role in textual health-information judgment requires further empirical validation. Specifically, it remains important to examine whether immediate corrective feedback is associated with more cautious judgment criterion and whether repeated exposure is associated with short-term, task-specific retention.

In summary, our current study utilized Signal Detection Theory (37, 38) to operationalize individuals' task-specific discriminability (*d*′) and their subjective misinformation susceptibility as decision criterion (*c*) when evaluating stroke-related information on the Chinese social media platform, Weibo. Across three experiments, we examined the joint effects of information prevalence, age, prior stroke-related experience, and corrective feedback on participants' fact-checking performance. We hypothesized that: (1) Middle-aged adults would exhibit lower task-specific discriminability (i.e., reduced discriminability) compared to younger adults (31); (2) Younger adults with prior stroke-related experience would demonstrate distinct information processing vulnerabilities (i.e., more liberal criteria) (32); and (3) The implementation of corrective feedback (Experiment 2 and Experiment 3) would be associated with more conservative criterion and improved task-specific discriminability at a 1-week follow-up (35, 36).

## 2 Experiment 1

### 2.1 Methods

#### 2.1.1 Participants

A total of 320 participants (*N*_*female*_ = 195) were recruited through an online survey platform, with the majority of targeted participants between 21–30 years old and 41–50 years old. This age selection was intended to align with both the typical age group of university students recruited in prior prevalence research (35) and the age group at which individuals begin to face elevated risks of first-time stroke onset (39). In total, 163 participants aged below 40 years old [*N*_*age*_[21,30] = 155] and 157 above 40 years old [*N*_*age*_[41,50] = 155] accomplished the task. Participants received RMB 4–5 (0.56–0.71 USD) for their participation. All the participants were given an informed consent form approved by the Human Research Ethics Committee of Central China Normal University (Protocol code: CCNU-IRB-20240325A). All the procedures performed in the studies involving human participants were in accordance with the ethical standards of the institutional and/or national research committee and with the 1964 Helsinki Declaration and its later amendments or comparable ethical standards.

#### 2.1.2 Materials and procedure

We downloaded the posts from all active users on the Chinese social media platform Weibo over the five-year period proceeding April 1, 2024. The posts were searched with targeted keyword combinations “(dispel) rumor/misinformation stroke” or “(dispel) rumor/misinformation apoplexy”. These various search combinations yielded approximately 740 posts. The downloaded posts were then screened to remove duplicates and those unrelated to the research context, resulting in 89 unique stroke-related posts. The remaining posts were labeled as either factual or misinformation based on their original context and were subsequently adapted into textual statements for the experimental tasks.

To evaluate the difficulty of these statements and select appropriate material for the main task, we conducted an online pilot test. A separate group of recruited participants were asked to judge whether each of the 60 candidate statements was true or false. A total of 60 participants participated in the task, receiving RMB 1.5 (0.2 USD) for their responses.

Based on the pilot judgment accuracy, factual and misinformation statements were classified into three difficulty levels: difficult, medium and easy. Examples are provided in Supplementary material. These items were then used to construct the regular- and low-factual-prevalence conditions, as summarized in Supplementary Table S2. In the regular-prevalence condition, the numbers of factual and misinformation statements were approximately balanced, with 26 factual statements and 24 misinformation statements. ^1^). In the low-factual-prevalence condition, there were 16 factual (i.e., targets/signal) statements and 34 misinformation (i.e., distractor/noise) statements. Thus, factual information served as the low-prevalence target, whereas misinformation served as the more frequent distractor.

Because the available number of eligible Weibo-derived posts was limited, some items overlapped between the two prevalence conditions. However, participants were recruited independently and randomly assigned to only one prevalence condition; therefore, no participant completed both conditions. A total of 160 participants were assigned to the low-factual-prevalence condition, and another 160 participants were assigned to the regular-prevalence condition. The text-based judgment task was administered as an online survey. Participants first reported their age range and then indicated whether they themselves or their family members had a stroke-related history before beginning the assigned task (The detailed number of participants for each condition can be found in Supplementary Table S3). The screened posts and the formal textual judgment material are publicly available at https://osf.io/n87q4.

### 2.2 Results

Participants' evaluations were documented as “correct” or “incorrect” selections and subsequently classified into four response types—hit, false alarms, miss, and correct rejection—based on the framework of Signal Detection Theory [SDT (38)]. Signal detection analyses were conducted using the package Psycho (40) in R. Detailed SDT calculations are provided in Supplementary material. Higher *d*′ value indicates better discriminability in distinguishing factual information from misinformation, whereas higher *c* values indicate a more conservative criterion for judging statements as true. Furthermore, to account for item-level variability, we also conducted a trial-level mixed-effects logistic regression as a robustness check for Experiment 1. The results are reported in Supplementary material.

**Figure 1:**
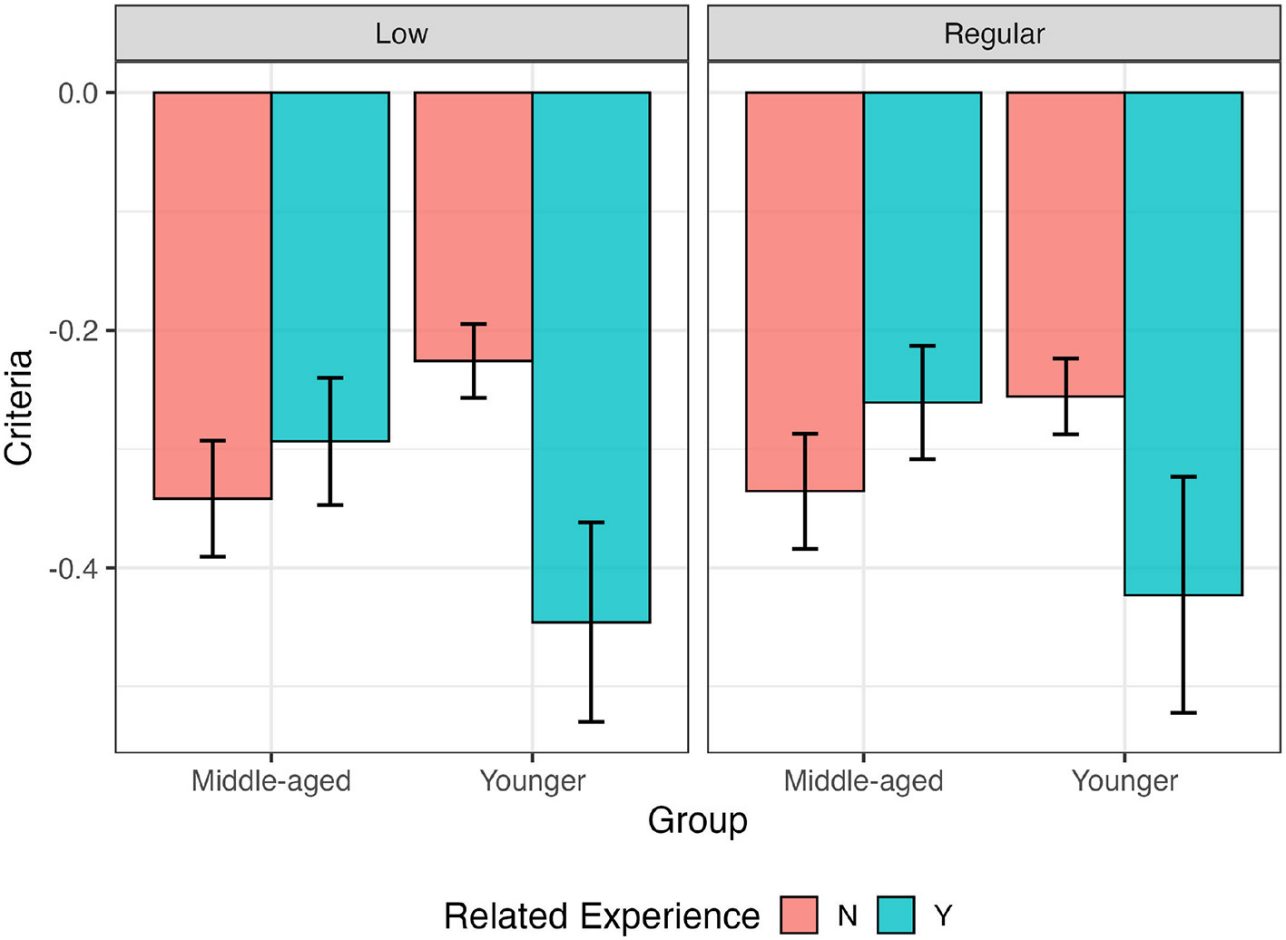
The average criteria for each age group in the regular- and low-factual-prevalence condition in Experiment 1, based on participants' prior stroke-related experience. The error bar represents one ± standard error.

**Figure 2:**
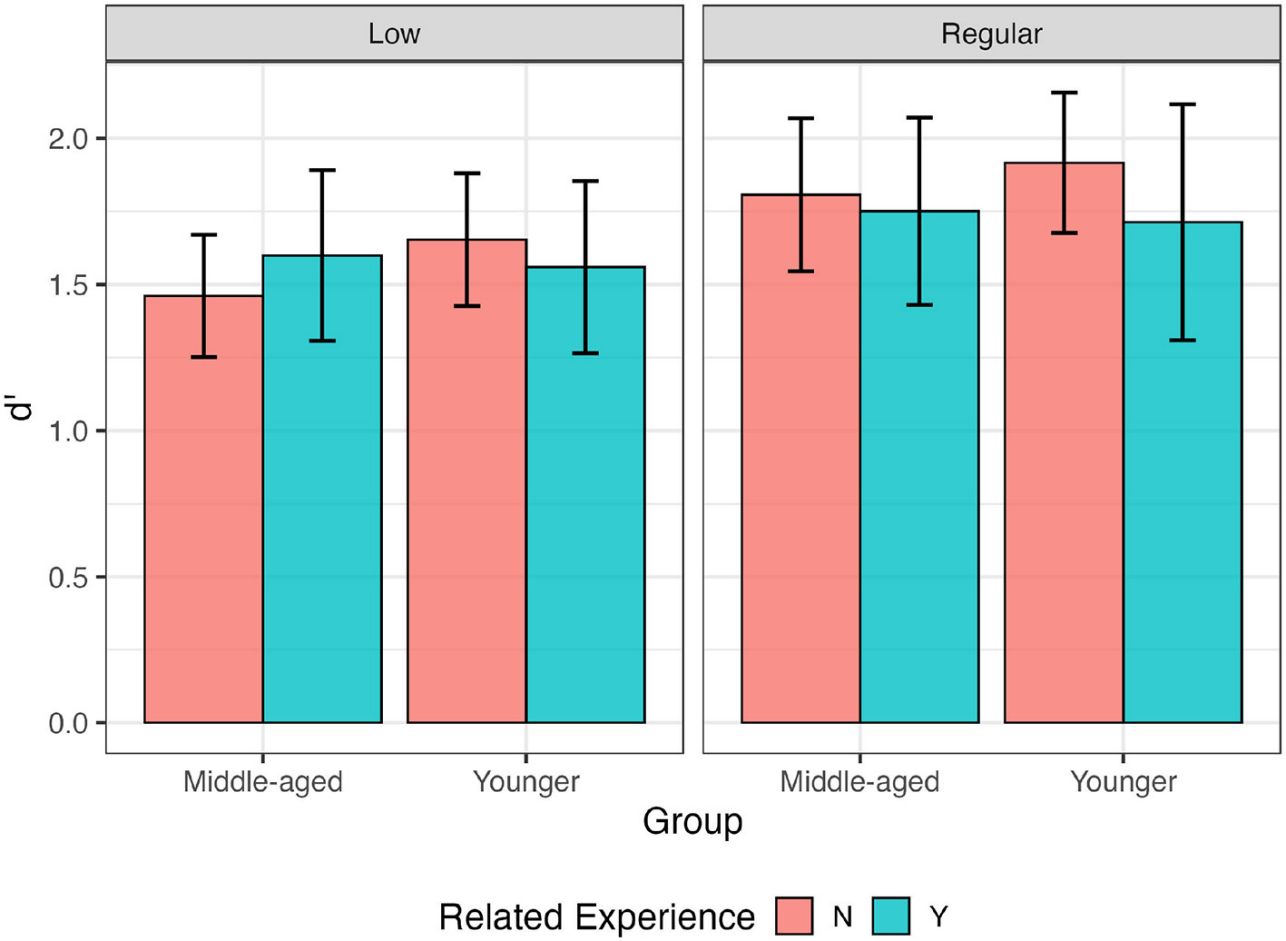
The average discriminability (d') for each age group in the regular- and low-factual-prevalence condition in Experiment 1, based on participants' prior stroke-related experience. The error bar represents one ± standard error.

The ANOVA results on decision criterion (*c*) revealed a significant interaction between the age group and prior stroke-related experience on the information judgment criterion (Figure 1)[*F*(1,312) = 11.83, *p* < 0.001, $\eta_{p}^{2}$ = 0.04]. Specifically, the interaction was driven by younger participants who had prior stroke-related experience experience and had more liberal criteria compared to participants who had not [*M*_*diff*_ = 0.19, 95%CI[0.05, 0.34], *t* = 3.56, *d* = 0.63, *p* = 0.003]. We also examine *d*′ as participants' discriminability in screening the factual from the misinformation statements (Figure 2). The results indicated that there was statistical significance in prevalence [*F*(1,312) = 12.03, *p* < 0.001, $\eta_{p}^{2}$ = 0.04]. Participants had statistically significantly worse performance in the low-factual-prevalence condition compared to the regular prevalence condition [*M*_*diff*_ = 2.28, 95%CI[0.10, 0.36], *t* = 3.47, *d* = 0.42, *p* < 0.001].

### 2.3 Discussion

In Experiment 1, we employed a fact-checking judgment task in which participants evaluated stroke-related statements derived from the social platform Weibo, determining whether each statement was accurate (i.e., factual) or inaccurate (i.e., misinformation). Contrary to our expectations based on classic prevalence-induced shifts in decision criterion (8, 36), we found no evidence that a low prevalence of factual information led participants to adopt more conservative or liberal decision criterion—reflected by increased missed errors or false alarms. Participants appeared to maintain a consistent baseline of information susceptibility (i.e., judgment criterion) across both regular- and low-factual-prevalence conditions. Moreover, no significant interaction between age and prevalence was observed, suggesting that age did not differentially influence susceptibility to prevalence effects in this digital health context (31). Despite the absence of an overall prevalence effect on overall decision criterion, we observed a critical vulnerability: younger participants with prior stroke-related experience—likely through family connections—were more predisposed to misclassify misinformation as factual (i.e., exhibiting more liberal criteria). This heightened misinformation susceptibility may stem from the emotional reinforcement of their personal experiences. Consistent with our previous research, non-experts may be more tolerant of false alarms than misses when evaluating uncertain health threats (12).

Furthermore, in contrast to previous public health surveys (32, 41), participants' prior stroke-related experience did not significantly predict their task-specific discriminability (*d*′). However, participants demonstrated greater discriminability under regular prevalence conditions, potentially due to an inherent belief bias toward factual content, as reflected in higher accuracy when identifying factual statements.

Unlike traditional real-world search tasks where corrective feedback is often absent (13, 14), the digital social media ecosystem frequently features expert fact-checking and platform-generated corrections. Given previous findings suggests that feedback could recalibrate decision criterion (35, 36), Experiment 2 aims to examine how corrective feedback influences individuals' online health information judgment, particularly under conditions where verifiable factual information is rare.

## 3 Experiment 2

### 3.1 Methods

#### 3.1.1 Participants

Another group of 50 participants (*M*_*female*_ = 30, *N*_*experience*_ = 21) participated in Experiment 2, which was conducted on the same online survey platform as Experiment 1, without a specific designated age range. As in Experiment 1, only participants' age ranges were recorded. The majority of participants were between 21 and 30 years old (*N* = 34), with additional 12 participants aged between 31 and 40 and 4 participants were older than 40 [*N*_*age*_[41, 50] = 3, *N*_*age*_[51, 60] = 1]. Participants received RMB 5 (0.7 USD) for their participation. All the participants were given an informed consent form approved by the Human Research Ethics Committee of Central China Normal University (Protocol code: CCNU-IRB-20240325A). All the procedures performed in the studies involving human participants were in accordance with the ethical standards of the institutional and/or national research committee and with the 1964 Helsinki Declaration and its later amendments or comparable ethical standards.

#### 3.1.2 Procedure

Participants were instructed to perform the task, indicating whether the presented stroke-related statements were correct or not, as the low-factual-prevalence condition in Experiment 1. Additionally, participants received a detailed explanation of why the statement was incorrect. For example: “Restricting sodium intake, regulating overall caloric intake, and maintaining balanced nutrition are inadequate for blood pressure management.” The presentation of statements was delivered in a random order for each participant.

### 3.2 Results

Participants' responses on the textual information were recorded, and we performed the same signal detection analysis as Experiment 1. Given that we only tested the feedback-present condition and most (45 out of 50) recruited participants were below 40, participants in the younger group in the low-factual-prevalence condition (*N* = 81, *N*_*experience*_ = 28) in Experiment 1 were included in Experiment 2 as the between-subjects design.

**Figure 3:**
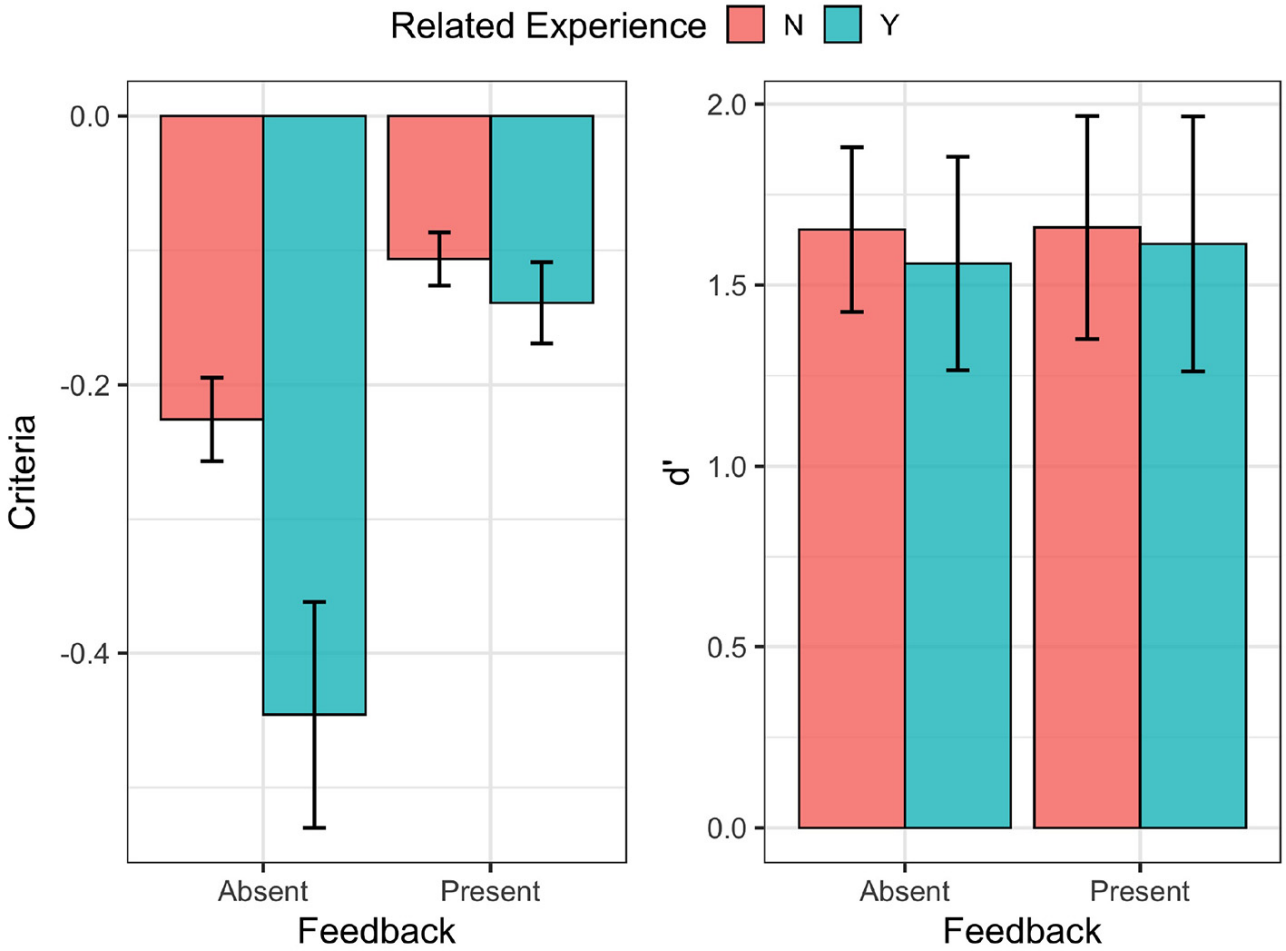
The average criteria and discriminability for each age group in Experiment 2, based on participants' prior stroke-related experience. The error bar represents one ± standard error.

Figure 3 describes the discriminability and the criteria for each group in Experiment 2. The ANOVA on decision criterion suggested that there was a main effect of the history [*F*(1,127) = 7.10, *p* = 0.009, $\eta_{p}^{2}$ = 0.05], participants who had prior stroke-related experience tended to exhibit more liberal criteria [*M*_*diff*_ = 0.13, 95%CI[0.03, 0.22], *t* = 2.67, *d* = 0.49, *p* = 0.009]. The results also suggested a main effect of the feedback [*F*(1, 127) = 20.21, *p* < 0.001, $\eta_{p}^{2}$ = 0.14]. Participants who received feedback were more conservative (indicated by a larger *c*) compared to participants who had no feedback [*M*_*diff*_ = 0.21, 95%CI[0.12, 0.31], *t* = 4.50, *d* = 0.49, *p* < 0.001]. There was also a significant interaction between the two factors [*F*(1,127) = 3.91, *p* = 0.05, $\eta_{p}^{2}$ = 0.03]. The interaction was led by the statistical difference between the feedback-present and feedback-absent conditions among the participants who had prior stroke-related experiences [*M*_*diff*_ = 0.31, 95%CI[0.11, 0.51], *t* = 4.14, *d* = 1.20, *p* < 0.001] whereas such difference was not found among participants who had no prior stroke-related experience [*M*_*diff*_ = 0.12, 95%CI[0.04, 0.28], *t* = 2.02, *d* = 0.47, *p* = 0.28]. Nevertheless, the ANOVA on discriminability (*d*′) failed to suggest a significant main effect on the prior stroke-related experience [*F*(1,127) = 0.44, *p* = 0.51] or the feedback [*F*(1,127) = 0.08, *p* = 0.77], nor was there an interaction between the two factors [(*F*(1,127) = 0.05, *p* = 0.82].

### 3.3 Discussion

Experiment 2 was conducted under the low-factual-prevalence performance condition to examine whether immediate corrective feedback was associated with a shift in judgment criterion. Consistent with prior cognitive research (36), participants in the feedback-present group showed more conservative criterion, compared with the feedback-absent group drawn from Experiment 1. This feedback-related difference was most evidence among younger participants with prior stroke-related experience. However, because Experiment 2 involved a between-sample comparison rather than a randomized feedback-control design, this finding should be interpreted as preliminary. Given that retention of stroke-related knowledge is critical for improving public health awareness and prevention practices (42), Experiment 3 aims to investigate whether repeated exposure to this corrective feedback could result in enhanced task-specific discriminability and judgment accuracy over time.

## 4 Experiment 3

### 4.1 Methods

#### 4.1.1 Participants

A total of 41 participants participated in Experiment 3, of which seven participants from the online data collecting platform in Experiment 2 returned for the tracking session in Experiment 3. Furthermore, we recruited another group of 37 participants from university students, of whom 30 completed both sessions. Participants received RMB 4 (0.56 USD) for their initial participation and RMB 6 (0.84 USD) for their subsequent accomplishment. Participants received RMB 10 (1.40 USD) in total for their participation. All the participants were given an informed consent form approved by the Human Research Ethics Committee of Central China Normal University (Protocol code: CCNU-IRB-20240325A). All the procedures performed in the studies involving human participants were in accordance with the ethical standards of the institutional and/or national research committee and with the 1964 Helsinki Declaration and its later amendments or comparable ethical standards.

#### 4.1.2 Procedure

Participants accomplished the same task using the same judgment material as in Experiment 2, where the low-factual-prevalence condition comprised 16 factual and 34 misinformation statements. Participants were invited to return for the tracking session after a minimum interval of 1 week (7–9 days) to assess the retention of stroke-related health knowledge.

### 4.2 Results

**Figure 4:**
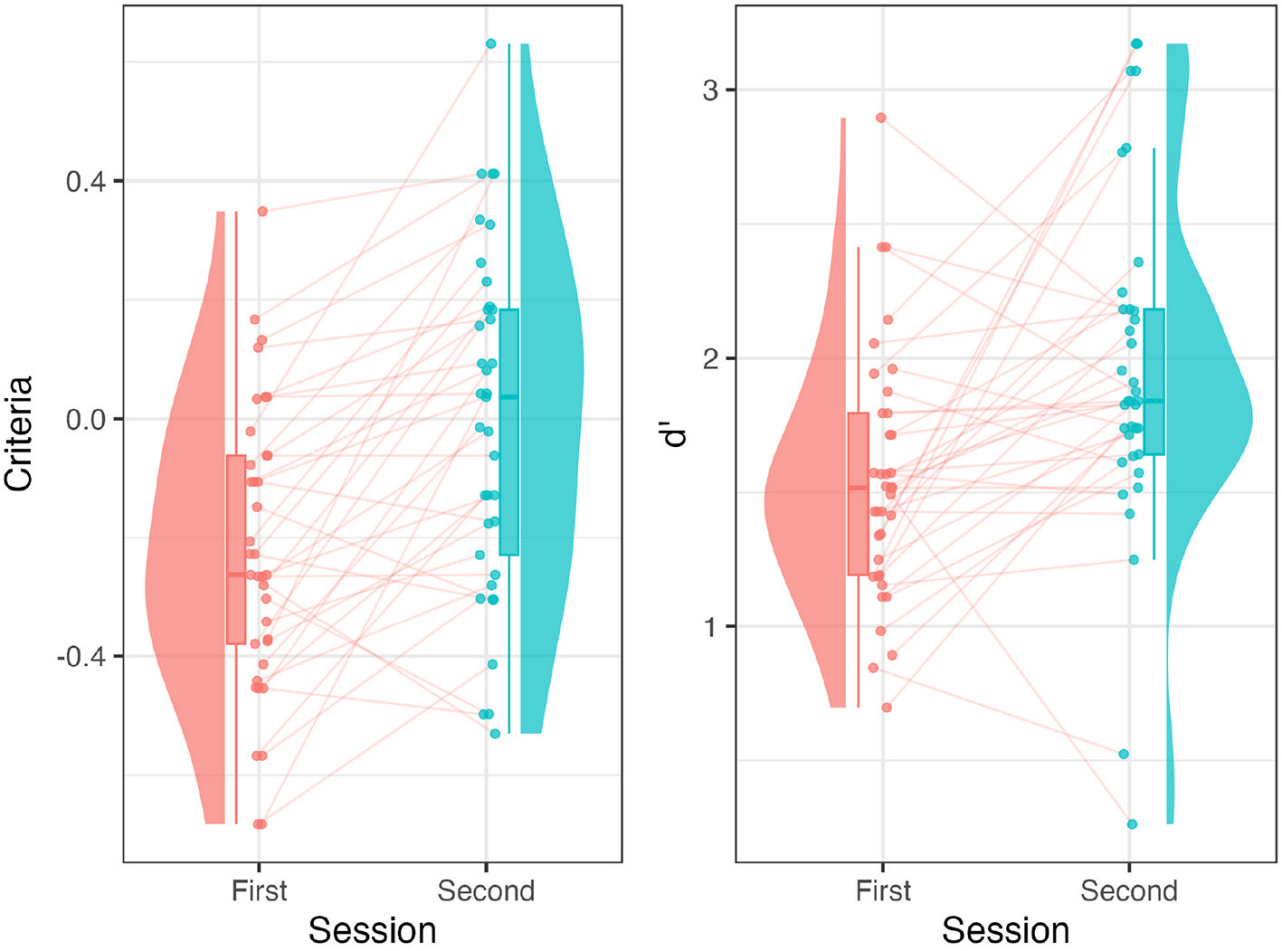
The average criteria and discriminability (d') for the first and second sessions in Experiment 3. The error bar represents one ± standard error.

Figure 4 describes the average criteria and discriminability in the two sessions in Experiment 3. Participants' performance was assessed using the paired sample *t*-test. The paired sample test indicated that participants in the second session (*M* = -0.005, *SD* = 0.29) exhibited more conservative criterion than those in the first session (*M* = -0.22, *SD* = 0.24), *t*(36) = 5.24, *p* < 0.001, *d* = 0.86. In addition, participants in the second session (*M* = 1.95, *SD* = 0.63) also exhibited superior discriminability relative to those in the first session (*M* = 1.54, *SD* = 0.46), *t*(36) = 3.52, *p* < 0.001, *d* = 0.58.

### 4.3 Discussion

The primary objective of Experiment 3 was to investigate whether the educational benefits of corrective feedback was associated with short-term, task-specific retention. The results indicated that, after a 1-week interval, participants showed significantly more conservative judgment criterion and higher task-specific discriminability when completing the same fact-checking tasks. Experiment 3 thus provided preliminary evidence that repeated engagement with corrective feedback was associated with more conservative judgment criterion and short-term task-specific improvement at a 1-week follow-up.

## 5 General discussion

Navigating the complex landscape of digital health information is crucial for making timely, high-stakes medical decisions, particularly for acute conditions like stroke. In the current research, we instructed participants to examine stroke-related health information to assess their task-specific discriminability on digital health information judgment. Contrary to our initial hypothesis regarding prevalence-induced shifts, participants maintained similar decision criterion across two distinct information prevalence situations in Experiment 1. Instead, a novel finding indicated that young adults with prior stroke-related experience, perhaps through familial connections, exhibited heightened misinformation susceptibility. Moreover, our Experiment 2 suggested that immediate corrective feedback was associated with a more conservative response tendency among younger adults, which may help reduce the likelihood of accepting misinformation as factual. Furthermore, our Experiment 3 monitored the retention of stroke knowledge. Our work provided preliminary evidence that exposure to the same corrective feedback were associated with enhanced task-specific discriminability (*d*′) as well as conservative criterion at a 1-week follow-up.

This study examined the impact of information prevalence on decision makers' evaluation of digital health information. Rectifying medical information poses a complex challenge, where both the misidentification of factual and the acceptance of misinformation statements yield significant public health impacts. As a result, decision makers need to exercise increased caution in establishing their criteria. Prior research indicated that those with lower educational attainment, as well as older adults, were more prone to believe and accept misinformation (43, 44). Nevertheless, the findings of Experiment 1 did not observe such an age-related vulnerability in our direct fact-checking task. This disagreement may arise from our differing task configurations; rather than assessing stroke knowledge by identifying risky factors (45). Notably, in alignment with prior studies indicating that bystanders and family members significantly influence the decision to seek assistance (19), our current research indicates that young adults with prior stroke-related experience demonstrated unique vulnerability, adopting more liberal criteria. However, one limitation of the current study is that the measure of prior stroke-related experience did not distinguish among different types of stroke-related experience, such as personal history, family history or general exposure to stroke-related information. Concerning that these forms of experience may influence health misinformation judgment through different mechanisms, future research should therefore directly measure and compare different forms of prior experience to clarify their distinct roles in stroke-related misinformation judgment, particularly when combined with alternative assessments of stroke-related knowledge (46, 47). Another limitation concerns the validation of the stimulus materials. In the current study, the *truth* status of stroke-related statements was classified based on the Weibo rumor-refutation context rather than through independent medical expert ratings. Moreover, because some posts were intended for public science communication, the scientific status of certain claims may involve nuance or ongoing debate. Future studies should include independent expert validation and more clearly distinguish established clinical facts from uncertain or context-dependent health claims.

The necessity for comprehensive population-level health education for stroke prevention and management is essential for decreasing the incidence of stroke as well as reducing stroke-related mortality and morbidity. A key distinction between our current research and previous studies health misinformation is that our Experiment 2 offered immediate and direct corrective feedback on participants' performance, whereas previous health research has often focused on macro-level strategies to combat and control the dissemination of misinformation (7, 48). Nevertheless, the feedback findings in Experiment 2 should be interpreted cautiously. The feedback-present group was compared with a feedback-absent group drawn from Experiment 1, rather than with a randomized feedback-absent control group within the same experiment. Although the same stimulus materials were used and items in Experiment 2 were randomly presented to participants, the comparison remained between samples; therefore, between-sample differences cannot be fully ruled out. Future studies should adopt randomized feedback-control designs with matched materials and feedback-control conditions. Consequently, subsequent studies may explore online fact-checking mechanisms in a greater fidelity social media context—particularly those that explore the effectiveness of various corrective messaging strategies (49) and the influence of source credibility (50). Furthermore, although previous work has demonstrated that television and newspaper campaigns have successfully improved public recognition of stroke symptoms (51), the modes of stroke-related information dissemination have shifted considerably with the rise of social media (45, 52). Thus the current digital landscape poses a significant challenge for identifying misinformation. Because our study focused exclusively on stroke-related misinformation on Weibo, future research should examine other platforms to explore how different digital environments facilitate the spread of false content. Additionally, identifying the characteristics of individuals most likely to share (mis)information will be crucial for developing targeted and effective intervention strategies. (53).

Crucially, our Experiment 3 provided preliminary evidence that participants may retain corrected information over a short interval in the present task context. DeLemos et al. (42), however, previously observed a significant decline in stroke knowledge following educational interventions, and only a limited number of participants modified their health behaviors. It is notable that in our current study, the follow-up interval was limited to 1 week, the same materials were used across sessions, and the task measured judgment performance rather than actual health behavior. Given that previous research also suggested that younger adults have often demonstrated greater baseline knowledge and better retention than their older counterparts (52), future research should examine whether corrective feedback produces longer-term knowledge retention and transfer (54), especially among older adults and in ecologically valid social media environments.

## 6 Conclusion

In conclusion, the research investigated how individuals evaluate stroke-related health information on social media, focusing on the roles of information prevalence, corrective feedback, prior stroke-related experience, and age group. Using Signal Detection Theory, we found participants did not show a significant prevalence-related shift in judgment criterion under the present text-based task. However, younger adults with prior stroke-related experience exhibited a more liberal judgment criterion, suggesting greater susceptibility to accepting misinformation as factual in this task. Furthermore, corrective feedback was associated with a more conservative judgment criterion, and repeated feedback was associated with improved task-specific discriminability at a 1-week follow-up. Ultimately, our research on individuals' judgment of social media health information provides useful insights for developing future strategies to identify, correct, and mitigate the spread of misinformation.

## Data Availability

The datasets presented in this study can be found in online repositories. The names of the repository/repositories and accession number(s) can be found in the article/Supplementary material.

## Footnotes

1 The initial design aimed for an equal distribution of factual and misinformation statements. However, one factual statement was incorrectly classified as misinformation during the pilot phase, resulting in a slight imbalance between the two categories.
