# Supplementary material for: Age differences in susceptibility to stroke-related health misinformation on social media: the role of prior experience and corrective feedback
Source: Front Public Health. 2026 Jun 18;14:1850524. doi: 10.3389/fpubh.2026.1850524 (PMC13323293; doi:10.3389/fpubh.2026.1850524)
Supplement: Supplementary file 1 [file Data_Sheet_1.pdf]

SUPPLEMENTARY MATERIAL

Examples of stroke-related information items varying in judgment accuracy in the pilot study.

- As people's pursuit of health increases, many businesses have introduced health-preserving recipes, with countless options specifically for lowering blood pressure. Limiting sodium intake, controlling total calories, and ensuring balanced nutrition can sufficiently manage blood pressure. (misinformation; judgment accuracy: 0.03)
- Blood pressure not only fluctuates daily but also experiences seasonal variations, typically being higher in autumn and winter, and lower in spring and summer. Therefore, it's advisable to consume more foods that help lower blood pressure during the winter months. (misinformation; judgment accuracy: 0.48)
- Occasional sharp pain in the chest that varies in location and lasts only a few seconds typically does not require excessive worry. (misinformation; judgment accuracy: 0.9)
- There is no scientific basis for using finger pressure on the "renzhong" point to raise blood pressure or improve breathing. (factual information; judgment accuracy: 0.58)
- When overeating, blood flow is limited to the digestive system, which can lead to reduced blood supply to the brain, making a person feel drowsy and sluggish. If this continues over time, it may even increase the risk of a stroke. (factual information; accuracy: 0.92)
- If you find yourself inexplicably tripping or falling frequently, it could indicate an issue with the brain's "executive function". (factual information; judgment accuracy: 0.98).

SIGNAL DETECTION THEORY MEASURES

In our current research, factual information was treated as the signal, whereas misinformation was treated as noise. Discriminability (d') and judgment criteria (c) were then calculated as follows:

Table S1. Signal detection theory classification in the factual-information judgment task.

Response	Factual information	Misinformation
True	Hit	False alarm
False	Miss	Correct rejection

$$d' = z(Hit) - z(FalseAlarm)$$

$$c = -\frac{1}{2}\left[z(Hit) + z(FalseAlarm)\right]$$

with higher d' values indicate better ability to distinguish factual information from misinformation and higher c values indicate a more conservative criterion for judging statements as true, whereas lower c values indicate a more liberal criterion.

Note. Prior experience refers to whether participants themselves or their family members had a stroke-related history.

Table S2. Summary of item difficulty levels in the regular- and low-factual-prevalence conditions.

Item type	Difficulty level	Regular prevalence	Low prevalence
Fact	Difficult	8	6
	Medium	9	5
	Easy	9	5
Misinformation	Difficult	7	12
	Medium	9	11
	Easy	8	11
**Total**		**50**	**50**

Note. The regular-prevalence condition included 26 factual statements and 24 misinformation statements. The low-factual-prevalence condition included 16 factual statements and 34 misinformation statements.

Table S3. Number of participants by age group, prior stroke-related experience, and prevalence condition in Experiment 1

Age group	Prior experience	Prevalence condition		Total
		Low-factual	Regular	
Middle-aged	No	49	48	97
	Yes	30	30	60
Younger	No	53	64	117
	Yes	28	18	46
**Total**		**160**	**160**	**320**

TRIAL-LEVEL MIXED-EFFECTS LOGISTIC REGRESSION IN EXPERIMENT 1

To account for both participant- and item-level variability in Experiment 1, we conducted an additional trial-level mixed-effects logistic regression predicting whether each statement was judged as true. The model included age group, prior stroke-related experience, prevalence condition, and their interactions as fixed effects, with random intercepts for participants and statement items.

The analysis revealed a significant main effect of prior stroke-related experience, $\chi^2(1) = 5.55, p = .02$, a significant main effect of prevalence condition, $\chi^2(1) = 79.98, p < .001$, and a significant prior experience $\times$ prevalence interaction, $\chi^2(1) = 7.31, p = .007$. Follow-up simple-effects comparisons were conducted using estimated marginal means to interpret the significant interaction. In the low-factual-prevalence condition, participants without prior stroke-related experience were less likely to judge statements as true than those with prior experience, odds ratio = 0.79, SE = 0.06, $z = -3.31$, $p = .001$. In contrast, this difference was not significant in the regular-prevalence condition. These results suggested that the association between prior experience and the tendency to judge statements as true was more pronounced under the low-factual-prevalence condition.
